# Data preparation and interannotator agreement: BioCreAtIvE Task 1B

**DOI:** 10.1186/1471-2105-6-S1-S12

**Published:** 2005-05-24

**Authors:** Marc E Colosimo, Alexander A Morgan, Alexander S Yeh, Jeffrey B Colombe, Lynette Hirschman

**Affiliations:** 1 The MITRE Corporation, 202 Burlington Road, Bedford MA 01730, USA

## Abstract

**Background:**

We prepared and evaluated training and test materials for an assessment of text mining methods in molecular biology. The goal of the assessment was to evaluate the ability of automated systems to generate a list of unique gene identifiers from PubMed abstracts for the three model organisms Fly, Mouse, and Yeast. This paper describes the preparation and evaluation of answer keys for training and testing. These consisted of lists of normalized gene names found in the abstracts, generated by adapting the gene list for the full journal articles found in the model organism databases. For the training dataset, the gene list was pruned automatically to remove gene names not found in the abstract; for the testing dataset, it was further refined by manual annotation by annotators provided with guidelines. A critical step in interpreting the results of an assessment is to evaluate the quality of the data preparation. We did this by careful assessment of interannotator agreement and the use of answer pooling of participant results to improve the quality of the final testing dataset.

**Results:**

Interannotator analysis on a small dataset showed that our gene lists for Fly and Yeast were good (87% and 91% three-way agreement) but the Mouse gene list had many conflicts (mostly omissions), which resulted in errors (69% interannotator agreement). By comparing and pooling answers from the participant systems, we were able to add an additional check on the test data; this allowed us to find additional errors, especially in Mouse. This led to 1% change in the Yeast and Fly "gold standard" answer keys, but to an 8% change in the mouse answer key.

**Conclusion:**

We found that clear annotation guidelines are important, along with careful interannotator experiments, to validate the generated gene lists. Also, abstracts alone are a poor resource for identifying genes in paper, containing only a fraction of genes mentioned in the full text (25% for Fly, 36% for Mouse). We found that there are intrinsic differences between the model organism databases related to the number of synonymous terms and also to curation criteria. Finally, we found that answer pooling was much faster and allowed us to identify more conflicting genes than interannotator analysis.

## Background

This article describes the preparation of training and test materials for a critical assessment of text mining methods in molecular biology (BioCreAtIvE) task 1B, which evaluated the ability of an automated system to generate the list of unique gene identifiers from PubMed abstracts for three model organism datasets [1]. We chose this task because we had available the sets of gene lists from the three model organism databases to serve as training and test materials. However, it was necessary to adjust these lists to our task. We had to make these adjustments because our guidelines for what to curate differed from all three databases and we were able to provide only abstracts, but two of the three datasets (Fly, Mouse) came from full text annotation. This meant that we had to modify the gene lists to reflect genes mentioned in the abstract – including, in some cases, genes that were not curated because they were only mentioned in passing. However, we felt that the abstract provided too little context to make these distinctions. Our data preparation therefore required removing genes from the database gene list that were not mentioned in the abstract (but only in the full text article), and adding in those that were mentioned "in passing" in the abstract. We used an automated procedure to quickly prepare large quantities of "noisy" training data.

Because the gene lists were used in an evaluation to score text-mining systems, we felt that it was particularly important to do a careful assessment of interannotator agreement on our test data, and, based on these findings, to improve the quality of annotation on the test set. This paper describes these experiments and an analysis of our process of generating the gene lists.

## Results

### Generation of gene lists

We were able to generate gene lists for three different model organisms, using data from their respective databases. This was done in several steps. First, we had to assemble a synonym list for each organism, using tables from the model organism database. This took about a week per organism. Then we had to manually adjust the gene lists by curating each abstract according to guidelines, which we developed (see additional data file 1). We estimate that an annotator was able to curate 250 abstracts in one week. An additional week was needed to assemble and verify the data. We also found it necessary to check the accuracy of our annotators by running an interannotator experiment. Three-way interannotator agreement on Fly and Yeast was reasonable (87% and 91% respectively) but the Mouse gene list had many conflicts (mostly omissions), which resulted in errors (69% interannotator agreement).

### Accuracy of gene lists

The interannotator experiment indicated that our gene lists contained errors. The cost of having an additional annotator look at each abstract would have been prohibitive. Therefore, we developed a secondary method to check for errors in our test data gene list. We used the participants' own results to check for errors. By pooling participant answers, we were able to quickly find additional errors. This only required one week for an annotator to check over the three sets of test abstracts (250 abstracts each) for errors. This method was fast, but it did not find all the errors; for example, it missed some of the errors detected by the interannotator experiment. Using this method, the Fly and Yeast annotations changed less than 1%; however, Mouse changed 8%. We conclude that our "gold standard" gene lists are of reasonable quality and well suited to evaluate information extraction systems, but probably still contain a small number of errors, particularly for the Mouse answer key.

## Methods

### Guidelines

We created a set of guidelines for the Gene List task (see additional data file 1 for the text of the guidelines). During the annotation of the development test data, the guidelines underwent several rounds of modifications. These changes were driven by questions raised by the annotators. This feedback helped to make the guidelines easier to follow and provide clear examples of problematic gene name questions. Additional questions arose during the annotation of the test data that led to changes in the guidelines. The annotators found this process very useful, especially the explicit examples that were developed.

The guidelines call for annotating explicit mentions of genes as well as gene mentions implicit in mentions of gene mutants, alleles, and products. Genes are required to come from the appropriate organism for the specific database (e.g., *Drosophila melanogaster *for Fly, *Saccharomyces cerevisiae *for Yeast, *Mus musculus *for Mouse) and must be identified by their unique identifier given in the model organism database synonym list. Any gene mentioned "in passing" should be included, even though these are often not included by the databases.

The mention of a gene has to be specific enough to identify the gene. Mentions of aggregates are allowed where this reference can be decomposed into specific genes. For example in the sentence, *Our data reveal that a prolonged OHT treatment, by increasing p44/42 MAPK activity, affects a key step in growth control of MCF-7 cells*, the gene identifiers include both the identifiers for p44 MAPK and p42 MAPK. Mentions of families of genes are included if they can be expanded to (a small set of) explicit gene mentions, as defined by the following rules:

• The mention of a gene that is part of a genome of many organisms and not attributed to a particular species is a sufficient mention for any of those species. For example, Histone H2A, if mentioned in a Mouse abstract, would count.

• From a single chromosomal location, each gene must be enumerated, and genes, which are explicitly enumerated, should be counted. For the latter, if the phrase was "The three SIX genes (SIX1-3)..." then *SIX1*, *SIX2*, and *SIX3 *should be added to the gene list. However, if the phrase was "The three SIX genes ...", then none of them would be added. This is because it is not clear, which three SIX genes are intended; for example, there might now be six SIX genes, while at the time the paper was written there might have only been three known SIX genes.

When orthologs are mentioned in an abstract for a given organism, but not directly associated with a specific organism, they are counted. For example, in the sentence *Galanin is a 29- or 30-amino acid peptide with wide-ranging effects on hormone release, feeding behavior, smooth muscle contractility, and somatosensory neuronal function*, we add the identifier for *Galanin *to the gene list, even though mouse is not mentioned in the abstract, because only one gene is mentioned and not a gene family. This gets tricky once paralogs are introduced, because they are gene duplications that might not be found in the specific organism being annotated [2]. For more detailed examples, see additional data file 1 for the Task 1B Guidelines.

### Lexical resources

For each organism, we provided participants with a synonym list that included:

• A mapping between gene symbols, gene names and the unique gene identifiers;

• A set of possible synonyms for each gene.

We derived the organism-specific lexical resources from the synonym and gene lists provided by each model organism database. We can see from Table 1 that the lexical resources for the organisms vary quite a bit, reflecting, in part, the variable number of genes and entries in each database (covering ESTs, multiple gene products per gene, and multiple species). Fly is predicted to contain only 13,525 genes, but there are an average of 2.8 synonyms per gene, whereas mouse is predicted to have 24,948 genes (ensembl predictions, ), with an average of 2.1 synonyms per gene. Yeast has the fewest predicted genes with ~6000 [3], and 1.9 synonyms per gene. Fly has the most ambiguity: 224 genes have over 20 synonyms, whereas neither Mouse nor Yeast has any genes with over 20 synonyms. The percent of entries with a single definition was 30% for Fly, 74% for Mouse, and 37% for Yeast (Table 1). Note also that the Fly resources contained genes for fly species other than just *D. melanogaster*.

### Generation of training and test data

To generate both training and test data for this task, we began with the resources available in three model organism databases (Fly [4,5], Mouse [6,7], Yeast [8,9]). We did this in three steps:

1. For each database, we created the synonym list (as described above), which included, for each gene, its unique identifier, the gene symbol and any synonyms listed in the model organism database.

2. Next, we extracted a list of PubMed IDs (pointing to the abstracts) and the associated gene lists for the *full text *articles in the model organism database.

3. We then "edited" these gene lists to make them correspond to the abstract.

For step 3, we did the editing in four stages, making use of the synonym lists generated in step 1 above

**Figure 1:**
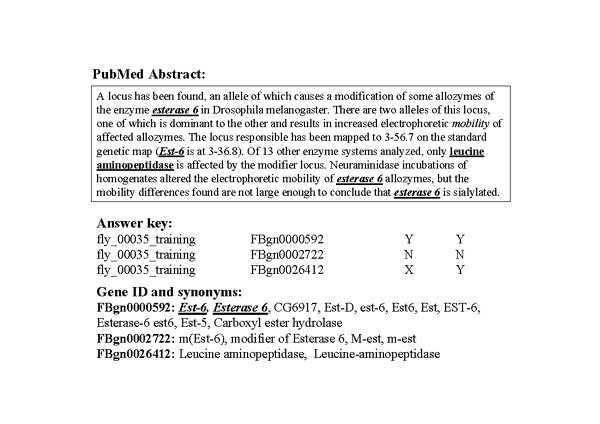
**Sample Abstract and "Noisy" Gene List. **Underlined and bold words in the PubMed Abstract are the genes were found in the text. The answer key consists of four columns. Column 1 is the file name; column 2 has the model organism unique database identifiers. Column 3 shows whether the gene was found automatically in the abstract (Y), not found and pruned (N), or added by hand (X). Column 4 shows the final set of genes in the answer key. This answer key shows that two genes were given by the database curators (FBgn0000592, and FBgn0002722); the first one was found in the abstract, the second one was not. The third gene (FBgn0026412) was found by our annotators based on the guidelines.

• Stage 1: We created a program that used the synonym list to search for mentions of each gene in the gene list for a given abstract. If it found a synonym in the abstract, it marked the gene present ("Y" in column 3, Fig. 1); if it did not find any synonym, the program marked it absent ("N" in column 3, Fig. 1). This enabled us to create large volumes of "noisy" (inexactly annotated) training data. We provided 5000 abstracts with gene lists for each of Yeast, Mouse, and Fly.

• Stage 2: For the development test set and the final test set, we had biologists with curation experience review and edit the lists generated in Stage 1. This is illustrated in Figure 1, which shows the gene list with an extra fourth column, reflecting the hand-checked entries, and a new row, representing a gene that was not on the database gene list but was added by the annotator. Initially, only one curator saw an abstract because of time constraints.

• Stage 3: We then evaluated the quality of the test data sets. We ran an interannotator agreement experiment between the initial curators, to determine (for at least a small sample set of 89 documents) how well the annotators agreed with each other in preparing the test key. We corrected the data set when we found that the initial curator made a mistake.

• Stage 4: As a further check, we also utilized the participants' own data using answer pooling. This led to the creation of a revised "gold standard" and a rescoring of all results.

For the hand-checking (Stage 2), the curators did two things to edit the gene list to correspond to the abstract: they removed genes that were mentioned in the full text, but not in the abstract; and they added back genes not listed either because of a matching error during the automated pruning, or because they were outside of the scope required by the specific organism database, but were within the scope of our guidelines. For example, the Mouse database does not curate information about adult gene expression. So, if a paper only contains information about adult gene expression for a gene, the information about that gene will not be curated, and the gene will not be listed. However, for the purposes of BioCreAtIvE, we decided that the abstracts contained so little text that it would be impossible to distinguish genes "mentioned in passing" from genes with sufficient data to merit curation. Thus, these genes, which might have not been curated by the databases, were added to the list.

### Evaluation of the data sets

Each database uses different criteria for what genes are curated and we only annotated the abstracts for BioCreAtIvE. Therefore, we expected many differences in comparing our final "answer key" with the gene lists from the model organism databases. Table 2 shows the results of this comparison. For Mouse, we added an additional 41% to the gene list, which were not on the original list (205 out of 495 total genes). The Yeast results were much closer, with only 12% added (75 out of 615 total genes). The Fly list had the fewest additions with 7% added (32 out of 431 total genes).

It was surprising to find so many genes mentioned in the Mouse abstracts that were not on the curated gene list for the corresponding full text article. This is presumably because the Mouse Database (MGI) is interested in genes with embryo expression, genes involved in mouse tumor biology, gene alleles, and evidence for gene ontology (GO) terms. MGI does not curate adult gene expression or genes mentioned in passing. So, we had to enter the additional "missing" genes in the hand annotation phase (205 genes). On the other hand, there were 795 genes listed for the full papers in MGI, and we only found 290 genes in the abstracts. Some of the genes from Mouse that we did not include in the gene list came from genomics papers. For example, two abstracts had 30 and 40 genes listed for them (lists of sequenced genes), but we found only a handful present in the abstract. In other cases, it was not clear whether the gene listed was a single gene or a gene family and as such, it was not added to the gene list. The remaining "missing" genes were presumed to be in the full text but not in the abstract. Overall, Fly and Mouse showed 25% and 36% respectively of genes listed in the database as being present in the abstract. This can be contrasted with Yeast (73%), which curates largely from abstracts unless there is something of particular interest, in which case curation is done from the full paper.

### Interannotator analysis

We performed an internal check on how well we annotated the abstracts. Each organism had a total of three annotators covering about 30 abstracts each, for a total of 89 abstracts from the test data set. These were the same annotators who worked on the generating the initial gene lists. Two annotators have a Ph.D. in biological sciences, one of which has experience as an annotator; the third annotator is a graduate student in biology. The results from this initial experiment of 3-way interannotator agreement are shown in Table 3. We analyzed these results to understand the sources of disagreement. A gene was marked as a disagreement if one of the three annotators disagreed. The most common mistake appeared to be just missing a term that was there. The second most common mistake was assigning a term that referenced a different species but was found in the target species. This was more of a problem for Mouse, since a human gene may be discussed in the same article as a mouse gene. If the genes share the same name, then it is necessary to be sure that the gene under discussion was really the mouse gene, not the human gene.

Mouse proved to be the hardest organism to annotate, with 28 conflicts out of 89 genes returned. Fly was easier to annotate, with 17 conflicts out of 129 genes returned, and Yeast the easiest, with only 6 conflicts for 64 returned genes. One interesting problem that arose was linking genomic loci or sequences to genes. For example, we needed to relate *M2-related DNA sequences are present on mouse chromosomes 4, 7, 12, and 13 *to identifiers like Rrm2, Rrm2-10, Rrm2-ps1, -ps2, and -ps4. These results demonstrated to us that it is important to conduct interannotator experiments. We were only able to check a small sample of the total abstracts, but based on this small study, we determined that our gold standard contained significant numbers of errors, especially for the Mouse data set.

### Improving the manual annotation: answer pooling

From the interannotator work, we already knew that the organism that posed the greatest problem was mouse. So we decided to use an answer pooling and selection method to check our gold standard. This was based on looking at genes that were marked as false positives for more than 75% of the participants (that is, we selected genes that the curators did not mark present but the participating systems did). We also looked at genes that all the participants failed to return (false negatives). We again found that Mouse had the most changes based on what the participants returned.

Using the 75% criteria, we found 5, 19, and 70 questionable gene references for Fly, Yeast, and Mouse, respectively (Table 4, False Positives). For Mouse, the annotations for 18 of the 70 genes were correct (these were indeed false positives by the participants). We also found two bad abstracts in that they were not annotated but given out to the participants. This mistake accounted for 6 more genes. Of the rest, 44 were not given on the model organism database list, i.e. not listed in the abstracts for the curator to see. Only 2 had been on the original database list, but by mistake, we marked them as not present. Both of these were in abstracts with a very big list of genes (~30 and ~40 genes on the list) in which most of the genes were missing from the abstract. Finally, we added an additional 7 genes when we were reviewing the abstracts in question. These additional genes were found by fewer than 75% of the participants.

The high number of conflicts in Yeast (19) was not expected, given the high level of interannotator agreement. However, 13 of the genes were correctly annotated by us (false positives by the participants). We correctly identified two genes, but because of formatting errors, these genes were not present in the gold standard. Four genes were not on the original database list and we missed them: the participants were correct in identifying them. Thus, as we found for Mouse, most of the ones we missed for Yeast were not on the original list.

For Fly, we only missed two genes. The other three genes, which all of the participants found were errors on their part (false positives). One false positive gene was scored as not present because it was from the wrong organism, *Staphylococcus aureus *PI-PLC. Another false positive was an interesting error, in that the systems returned the shorter of two gene names; they picked "Histone H2A" over "Histone H2A variant". The term "Histone H2A" was present in the abstract, but in reference to other organisms and as a family. The last false positive is more perplexing, in that the participants' systems picked a gene name that had a partial match over one that matched exactly: they picked DERK over DER for the reference of *the receptor tyrosine kinase DER*.

We next looked at the genes that all of the participants missed, which were 14 for Yeast, 15 for Fly, and 43 for Mouse (Table 4, False Negatives). For Mouse, 34 of the candidate genes were genuine misses by the participants; i.e. the gold standard was correct. Nine were mistakes by us. A major cause of these misses seemed to be in converting between different forms of a name. For example, gene-1 and gene-2 vs. gene-alpha and gene-beta or adding a number or symbol to a gene name which lacks one because at the time the article was written there was only one gene. Another general problem was the problem of conjoined names, for example, *MT-I and -II *or *designated Otf-3a through Otf-3h*. There was one case of a prefix problem. In this case, the prefix "pro" was added to signify that it is modified to make the final protein.

For Fly, 12 of the candidate genes were correct and 3 were errors by us. In two cases, an allele designation probably hindered detection by the participants. The correct way to mark an allele for flies is to superscript it. However, in these two cases this was not done probably because of the use of ASCII text. So, *flr3 *in the abstract should have been *flr*^3 ^and there is no *flr3 *gene. Finally for Yeast, 11 of the candidate genes were correct and 3 were errors by us. It seems once again that getting conjoined names was a problem. An additional problem was handling different spellings and word order for complex proteins, such as *nad dependent isocitrate dehydrogenase *(as it appeared in the synonym list) vs. *NAD-specific isocitrate dehydrogenase *(used in the abstract).

Overall, our most common annotation mistakes were just simple errors, such as missing a mention, formatting, copying and pasting errors, and relying too heavily on the model organism list for each article. In the last case, we would say that a gene was present because the database said it was there and we found one that matched. However, in some cases, the database only put down one family member and there were several more. Since our guidelines said that we would only count explicitly enumerated gene families, we were wrong to have annotated a gene family name, as opposed to the explicit gene. This round of careful checking greatly improved our gold standard, especially for Mouse.

### Overall changes in the gold standard

Using the above methods, we found that some of our gold standard annotated data changed significantly. The most drastic was Mouse, where we found an F-measure of 0.920 measuring the original "gold standard" against the revised gold standard (Table 5). Fly had 0.993 and Yeast had 0.987 for F-measures. Our precision for Mouse was fairly good (0.966), but our recall (0.879) was the lowest for all three organisms.

**Figure 2:**
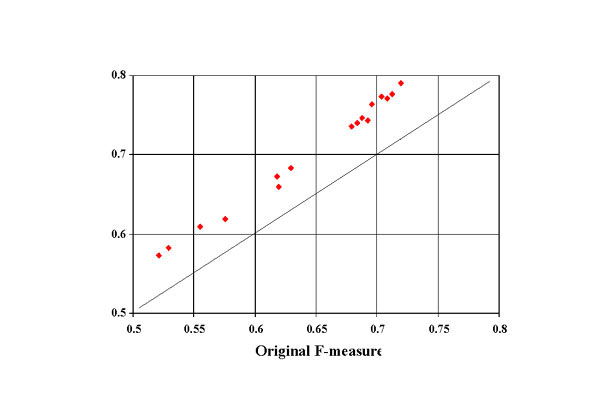
**Changes in Participant's Mouse F-measures. **Graph showing the differences between the participant's original F-measure and their final F-measure.

We also looked at how these changes affected relative rankings of systems (Figure 2). Looking at just Mouse F-measure, the overall standings did not change much. Out of 15 submissions, 3 submissions changed relative rankings based on F-measure. This did result in a change in third place. However, there was no change when looking at precision. There were four submissions that changed in recall; two of these were also ones that changed positions in F-measure.

## Conclusion

We have found that generating a list of genes found in abstracts is a complex problem. We concluded that it is important to have well-defined guidelines, but even with guidelines, it is critical to check the annotators against each other for consistency.

We found that some organisms were harder to annotate than others depending on how the original gene lists were developed. There are intrinsic differences between the model organism data sets, which reflect the differences in how each database curates (full text vs. abstracts), differences in what each database is interested in curating (such as specific or general information on genes), differences in lexical resources, and differences in the scientific communities writing the papers. Compared to the three organism databases, our Task 1B annotation guidelines were very liberal, which accounts for most of additions to our gene lists. We made the least number of mistakes for Fly. This could be because we had to add the least number of genes to the Fly gold standard. Also, it was surprising that we made more mistakes with Yeast, considering that its lexicon was by far the simplest and smallest. It is unknown why we had to add more genes to Yeast than to Fly.

We also concluded that abstracts alone might be a poor resource for identifying genes in a paper (see Table 6): for Fly, only 25% of genes on the full text gene list were mentioned in the abstract. For Mouse, the figure was 37%. On the other hand, for Yeast, which was curated largely from abstracts, the figure was 73%.

This experiment shows that we were able to prepare data sets that consisted of lists of gene identifiers mentioned in abstracts. We were able to use the model organism gene lists from full text articles as a starting point and adapt these to our task. The automated adaptation produced a relatively high precision training data set (results reported in [1]), with a number of "missed" genes that had to be added manually. Our interannotator agreement experiments showed that these missed genes were the main source of error. Our error varied considerably, with mouse abstracts having the greatest number of missed genes.

The cost of validating our methods was significant: we estimate that it took about a person-week to generate each 250 abstract test set. Overall, it took about half that much time to do run the various answer pooling experiments. This raises the issue of whether it would be preferable (and cheaper) to use full text data and the original model organism gene lists for future experiments. This would pose a more difficult, but more realistic, task for the automated systems, since they would have to process full text articles and take into account the criteria for gene curation, which differ among model organism databases.

## Authors' contributions

MEC annotated Mouse and Yeast, collected the interannotator and participants' results, and analyzed data. AM annotated Yeast, created the guidelines, obtained and created the lexical resources for Fly, and obtained and organized the corpus of abstracts. AY obtained and created the lexical resources for Yeast and Mouse. JC annotated Fly. LH is the principle investigator for the MITRE BioCreAtIvE effort.

## Supplementary Material

Additional File 1 **Guidelines for BioCreAtIvE Gene List Evaluation **Guidelines for what genes should be listed in this Task 1B Gene List task. Click here for file

## Figures and Tables

**Table 1:** Statistics for Lexical Resources

Lexical Stats	# Entries	# Synonyms	Ratio	Maximum # of Synonyms per Gene	# with One Definition	Predicted # of genes
Fly	35,971	99,501	2.766	96	10,863	~14,000
Mouse	52,595	109,516	2.082	19	39,135	~25,000
Yeast	7,929	14,756	1.861	10	2,955	~6,000

**Table 2:** Composition of Gene Lists

		Manually Found in Abstracts				
		
Organism	Number of Genes on Database List	Genes on DB List	% on DB List	Genes Added to List	% Total Manual Genes	Total Genes
Fly	1571	399	25	32	7	431
Mouse	795	290	36	205	41	495
Yeast	737	540	73	75	12	615

Percent of the genes on the database (DB) list describes what percentage of the genes we found in the abstracts that were on the lists given from the databases. Percent total manual genes are the percents of the genes we added out of the total genes found.

**Table 3:** Interannotator Agreement Experiment.  A gene was marked "disagree" if one out of the three annotators disagreed.

Organism	Genes Annotated	Disagree	% Disagree
Fly	129	17	13%
Mouse	89	28	31%
Yeast	64	6	9%

**Table 4:** Conflicting Genes from Answer Pooling

Organism	False Positives		False Negatives	
	
	# Candidates	# Correctly Annotated	# Candidates	# Correctly Annotated
Fly	5	3	15	12
Mouse	70	18	43	34
Yeast	19	13	14	11

**Table 5:** Changes in the Gold Standards

Metric	Scores of the Original gold-standards		
	
	Fly	Mouse	Yeast
F-measure	0.993	0.920	0.987
Precision	0.991	0.966	0.989
Recall	0.995	0.879	0.985

**Table 6:** Final Comparison of Found Genes

Organism	# Genes on Database List	# Found from List	% Overlap with Database	Total # Additional Genes Added	%Added Genes of Total	Total Genes
Fly	1571	399	25.4	34	7.9	429
Mouse	795	290	37.1	271	49.8	544
Yeast	737	540	73.3	84	13.7	613
