# Supplementary material for: Data preparation and interannotator agreement: BioCreAtIvE Task 1B
Source: BMC Bioinformatics. 2005 May 24;6(Suppl 1):S12. doi: 10.1186/1471-2105-6-S1-S12 (PMC1869005; doi:10.1186/1471-2105-6-S1-S12)
Supplement: Additional File 1 — Guidelines for BioCreAtIvE Gene List Evaluation Guidelines for what genes should be listed in this Task 1B Gene List task. [file 1471-2105-6-S1-S12-S1.doc]

# Guidelines for BioCreAtIvE Gene List Evaluation

## Sub Task 1.B

Create the list of unique genes that are mentioned in an abstract referenced by a model organism database (Fly, Mouse, and Yeast). These mentions will include explicit mentions of genes as well as gene mentions implicit in mentions of gene mutants, alleles, and products. Genes must come from the appropriate organism for the specific database (e.g., Drosophila melanogaster for Fly, Saccharomyces cerevisiae for Yeast, Mus musculus for Mouse) and must be identified by their unique identifier in the database.

This approximates the curator’s task of listing the set of genes relevant to the appropriate model organism database. It may require expert knowledge and use of context to determine which unique identifier to associate with a gene mention in the case of an ambiguous name that can refer to several different genes (e.g., in FlyBase, “clk” is the gene symbol for the “Clock” gene but it also is used as a synonym of the “period” gene). This is an unavoidable aspect of the real task.

**Guiding principles/goals:**

1. Identify the key genes mentioned in the abstract, in normalized form.
2. Any mention of a gene counts, including those mentioned in alleles, mutants, and gene products.
3. Even genes mentioned “in passing” should be included, although these are often not included by the curators (who are primarily interested in curating genes for which there is some significant finding in the paper).
4. The mention of a gene must be specific enough to identify the gene; mentions of aggregates will be allowed.

**Examples associated with the principles:**

Below, gene mentions are highlighted in ***bold italics.***

1. Explicit mention of a gene name

"Mutations in the Drosophila gene ***pavarotti*** result in the formation of abnormally large cells in the embryonic nervous system."

"Dependence on the intensity of Drosophila virilis sexual behavior on age-related differences was studied. Most active proved to be those age periods which correlated with the time of S-esterase elevated activity." [S-esterase is a Drosophila virilis gene and does not count as a mention of a Drosophila melanogaster gene]

1. Mention of a mutant, allele, transcript or a product in such a way there is clear association with a single gene:

"In some of these mutants (***fat***, ***lgd***, ***c43***, ***dco***) the overgrowing tissue is hyperplastic..."

"A locus has been found, an allele of which causes a modification of some

allozymes of the enzyme ***esterase 6*** in Drosophila melanogaster."

3. Example of a gene mentioned in passing:

"Fast and slow electrophoretic variants of this protein map between the markers ***vin*** and ***gs***, at 36--37 on chromosome 3."

4a. Protein complexes named as an aggregate group of polypeptides encoded by different genes do not constitute a mention of a particular gene, but if the individual components, traceable to specific genes, are discussed, those will count as mentions of genes.

"We have analyzed the cis-acting regulatory sequences of the Drosophila melanogaster ***Rh2*** gene that encodes the protein component of a rhodopsin which is expressed ..." [Note that “a rhodopsin” is not counted as a mention because it is not specific.]

4b. Aggregates should count as distinct mentions.

"Our data reveal that a prolonged OHT treatment, by increasing ***p44/42 MAPK*** activity, affects a key step in growth control of MCF-7 cells” [This mention should generate identifiers for p44 MAPK and p42 MAPK].

4c. Mentions of families of gene names count as mentions in a few cases

- 1. The mention of a gene which is part of the genome of many organisms and not attributed to a particular species is a sufficient mention for any of those species.
  2. Across functional/structural grouping the mention counts only if the set is small and enumerated (see 4b. above).
  3. From a single location chromosomal location, genes must be mentioned separately.

"... homologous to the serine protease family of enzymes." [This does not count as a mention. The serine proteases are not linked to a single chromosomal location or described as an enumerated set].

## Materials:

The following will be provided for each model organism.

1. A lexical resource [*LEXICON*] (e.g. mouse_synonyms.list) consisting of the list of unique identifiers for the genes of each organism, along with the canonical gene name, symbol, and a list of some common synonymous variants, as gleaned from materials available in the model organism database.
2. For each organism, a training set of approximately 5000 abstracts [*ABSTRACTS*] (e.g. mouse_03334_training.txt), each with a “partially cleaned” gene list [*AUTOCLEANE_GENE_LIST*] (e.g. mouse_03334_training.gene_list). This list is generated from the original gene list in the model organism database [*DB_GENE_LIST*]. The cleaning process is explained below.
3. A handful of abstracts with a hand corrected gene list [*HANDCLEAN_GENE_LIST*] (e.g. fly_00035_devtest.gene_list).
4. A scoring script written in Perl which will automatically score the results of automatic systems.

Testing procedure:

1. The text of approximately 250-500 abstracts per model organism will be made available for download a few days prior to the deadline to turn in results.
2. Participants have several days to create a gene list for each abstract, together with an indication of a supporting 'mention/evidence' for each gene (a text string corresponding to a mention of the gene from the abstract).
3. The output format is illustrated below, in the section on Testing Data. It will consist of a list of the genes found (one line per gene per abstract, with supporting evidence).
4. The mechanism of submission will be explained to all participants in a subsequent email.

**Gene Lists:**

The model organism databases use either abstracts or full text articles in the curation process. Where full text articles are used, the gene lists in the database reflect the genes mentioned in the entire article. The genes listed may or may not be mentioned in the *abstract*. In addition, each database uses different criteria for inclusion of a gene in the list. Therefore, a gene may be present in the abstract but not mentioned in the gene list. To provide gene lists that correspond more accurately to what is mentioned in the abstracts, we have put in place an automated cleaning process that aligns the gene lists with the text from the abstracts. This cleaning process is noisy, and users are warned of the following possible inconsistencies:

1. A gene mentioned just “in passing” may not appear in the *DB_GENE_LIST*, and hence, it will not appear in the *AUTOCLEAN_GENE_LIST*. This absence could cause what appears to be a false positive, if an automated system correctly detected such a gene in the abstract.
2. The cleaning process may mistakenly leave in a gene that has, as one of its synonyms a commonly occurring word (e.g., “for”), even where this gene is in fact not mentioned in the abstract. This could cause a false negative.
3. The cleaning process may fail to find a gene mention in the abstract that is a slight variant of something in the lexicon – and thus erroneously remove the gene in producing the *AUTOCLEAN_DB_LIST*. This could cause an apparent false positive.
4. We have prepared gene lists *HANDCLEAN_GENE_LIST* for a small set of approximately 150 abstracts for FlyBase to determine how “noisy” the *AUTOCLEAN_GENE_LIST* set is. For FlyBase, the automated cleaning removed approximately 2/3 of the genes from *DB_GENE_LIST.*  Overall, we measured a precision of 0.88 and a precision of 0.77 for *AUTOCLEAN_DB_LIST* against the *HANDCLEAN_GENE_LIST.*

We plan to provide additional small hand-cleaned gene lists for the other model organism databases.

The gold standard gene lists for the test data will be prepared by hand.

## Lexical Resources:

We will provide a synonym list for each organism. The format of this list is as follows:

- - Each gene has its own line in the document.
  - The unique identifier for the gene in the organism specific database is the first element in each line.
  - The rest of the line is a tab-separated list (i.e. horizontal tab, 0x09, &#009;) of alternate naming forms used. It is not an exhaustive list of the names used, but can include alternate spelling and capitalization forms. Often a short 'gene symbol' is the first entry in the list which may be followed by a longer 'gene name' and then finally alternate forms used to refer to the gene. These names are NOT unique and can be found in other gene lists.
  - In some cases, the databases use specific formatting for indicating special characters such as Greek letters (for example &agr; is used to refer to lower case alpha in FlyBase and there is special formatting for sub and superscripts). We have left these formatting indicators in the synonym lists as well as including a synonymous form that either turns the formatting character into some ASCII representation (e.g. &agr; -> alpha) or removed them if there was no clear ASCII translation (e.g. <sub>). Note that a name may consist of multiple words; that is, there may be embedded white space, hyphens, dashes, slashes and commas in the name.

An excerpt from the Drosophila melanogaster gene synonym list follows:

FBgn0033436 CG12930

FBgn0033431 CG1827

FBgn0014865 Mtk Metchnikowin CG8175 Metch metchnikowin metchnikowin-1

FBgn0033439 CG1773

FBgn0000592 Est-6 Esterase 6 CG6917 Est-D EST6 est-6 Est6 Est EST-6

FBgn0003510 Sry-&agr Serendipity &agr; Sry-alpha Serendipity alpha

An excerpt from the Mouse gene synonym list follows:

MGI:2150994 Drb2 dopamine receptor binding 2

MGI:1354954 Nt5c 5',3'-nucleotidase, cytosolic Umph-2 Umph2 Dnt1 Dnt

MGI:97172 Mt2 metallothionein 2 MT-II Mt-2

### Important Notes:

1) The mouse entry "MGI:1354954" has the entire string "5',3'-nucleotidase, cytosolic" as a single synonym.

2) Many of the entries with unique identifiers are not traditional protein coding genes, such as short RNA coding regions, chromosomal loci, etc. We may exclude these from the evaluation later, but for now they are "fair game".

3) There are 4 pairs of adjacent yeast genes which in some strains share an open reading frame (ORF) and in others have an intervening ORF. We have removed these genes from the synonym list and papers known to contain discussion of these genes from the pool of abstracts. The accession numbers for these genes are: S0006196 (YPL275W, FDH2), S0006197 (YPL276W, FDH2), S0001432 (YIL170W, HXT12), S0001433 (YIL171W, HXT12), S0001811 (YKR103W, NFT1), S0001812 (YKR104W, NFT1), S0003939 (YLL016W, SDC25), and S0003940 (YLL017W, SDC25). For the purposes of this evaluation, we are 'ignoring' these genes.

## Examples:

The task involves finding the list of genes (unique identifiers) with mentions in a document (MEDLINE abstract). The training data will consist of a set of ASCII files, each containing a single abstract along with a series of associated short files which contain a list of the genes curated for the reference associated with that abstract and an indication of whether or not any of the gene name alternatives obtained from the synonym lists was found in that abstract.

A file named "fly_00035_training.txt" would contain the following text:

A locus has been found, an allele of which causes a modification of some

allozymes of the enzyme esterase 6 in Drosophila melanogaster. There are

two alleles of this locus, one of which is dominant to the other and

results in increased electrophoretic mobility of affected allozymes. The

locus responsible has been mapped to 3-56.7 on the standard genetic map

(Est-6 is at 3-36.8). Of 13 other enzyme systems analyzed, only leucine

aminopeptidase is affected by the modifier locus. Neuraminidase

incubations of homogenates altered the electrophoretic mobility of

esterase 6 allozymes, but the mobility differences found are not large

enough to conclude that esterase 6 is sialylated.

A file entitled "fly_00035_training.gene_list" would contain the following tab delimited lines:

fly_00035_training FBgn0000592 Y

fly_00035_training FBgn0002722 N

The first element is simply the name of the training file associated with that entry (it is repeated on each line in case the gene_list files are concatenated). The next element is the unique identifier of a gene indicated by the database curators as being associated with that reference (either the abstract or the associated full text research article). The final entry is whether or not any known synonyms for that gene were found in the abstract (Y/N).

If the abstract has been examined by hand and all mentions of genes determined based on the guidelines, then an additional file "fly_00035_devtest.gene_list" would exist and would contain the following tab delimited lines:

fly_00035_training FBgn0000592 Y Y

fly_00035_training FBgn0002722 N N

fly_00035_training FBgn0026412 X Y

In this case, the new forth column indicates whether or not a gene mention was found during a close scrutiny of the abstract by a domain expert. The third line is an additional entry that refers to the gene "leucine aminopeptidase" which was not on the list created by the database curators, but a mention was found in the abstract. In this case the third element is an "X" which indicates that this gene was not on the original list of genes created by the database curators and associated with this document.

## Testing Data:

The data used to evaluate the systems will be very similar. A collection of 250-500 abstracts for each organism will be provided. The task will be to create a list of the unique identifiers of the genes mentioned in each abstract. Using our previous example, if this abstract were to appear in the test data, it might be named "fly_00035_testing.txt". It would appear as:

A locus has been found, an allele of which causes a modification of some

allozymes of the enzyme esterase 6 in Drosophila melanogaster. There are

two alleles of this locus, one of which is dominant to the other and

results in increased electrophoretic mobility of affected allozymes. The

locus responsible has been mapped to 3-56.7 on the standard genetic map

(Est-6 is at 3-36.8). Of 13 other enzyme systems analyzed, only leucine

aminopeptidase is affected by the modifier locus. Neuraminidase

incubations of homogenates altered the electrophoretic mobility of

esterase 6 allozymes, but the mobility differences found are not large

enough to conclude that esterase 6 is sialylated.

The results for the entire set of texts are submitted in a single file, with three columns. The first column will be the file id, the second the normalized form of the name (the unique identifier), and the third line is a piece of extracted text (with no linebreaks) which corresponds to the mention as it appears in the text. A correct submission for this file would include these lines:

...

fly_00035_testing FBgn0026412 leucine aminopeptidase

fly_00035_testing FBgn0000592 Est-6

...

Submissions will be scored on obtaining the correct set of unique genes; the third column containing the extracted text must be present for the entry to be counted. We will develop some semi-automated scoring tools to check the (the “evidence”) in this field for the final test data.

The submissions should be automatically generated using a "frozen" set of system parameter settings. The testing data should be input into the system to be evaluated without a human examining the data (input text files) and the output should also be submitted to us without any human observing the results or modifying the system in any way.

## Information for Annotators:

The annotation process is as follows: the abstract and its associated gene list are “harvested” from the model organism database. For noisy cleaning, the abstract and the list are run through a filter to remove any entries missing from the abstract. For a small sample of the training data, and for all of the test data, these results are then carefully manually verified and corrected by human expert annotators.

To do this, we have developed a third form of data, to aid the annotator. Below is an example of the data provided to the annotator. The text and annotators are given in XML, because we will be using an XML-based annotation tool.

For each text, the file contains XML with the PubMED ID (pmid), the gene list and the text.

The gene list (DBGENELIST) consists a term (DBGENE) for each unique gene listed by the model organism database. The gene entry consists of the unique identifier (DBID), followed by two attributes with “Yes/No” values and a third field with the synonyms for that gene. The first term is “AF” for automatically found in the abstract. A “yes” indicates that the automatic procedure found one or more mentions of the listed gene in the abstract; a “no” indicates no mention was found. Following this is the “HF” field, for entries found by hand. In the input to the annotator, this field is marked with a “?” and must be changed to a Y or N value during the annotation process. Following these attributes is the list of known synonyms and alternate forms for the gene. This is provided as a convenience to the annotator, so s/he does not have to spend time looking up each gene to find its synonyms.

<DOC pmid="110315">

<DBGENELIST>

<DBGENE DBID="FBgn0000592" AF="Y" HF="?"/><!--Est-6, Esterase 6, CG6917, Est-D, EST6, est-6, Est6, Est, EST-6, Esterase-6, est6, Est-5, Carboxyl ester hydrolase-->

<DBGENE DBID="FBgn0002722" AF="N" HF="?"/><!--m(Est-6), modifier of Esterase 6, M-est, m-est-->

</DBGENELIST>

<TXT>

A locus has been found, an allele of which causes a modification of some

allozymes of the enzyme esterase 6 in Drosophila melanogaster. There are

two alleles of this locus, one of which is dominant to the other and

results in increased electrophoretic mobility of affected allozymes. The

locus responsible has been mapped to 3-56.7 on the standard genetic map

(<gn ID="FBgn0000592">Est-6</gn> is at 3-36.8). Of 13 other enzyme systems
analyzed, only leucine aminopeptidase is affected by the modifier locus.
Neuraminidase incubations of homogenates altered the electrophoretic
mobility of esterase 6 allozymes, but the mobility differences found
are not large enough to conclude that esterase 6 is sialylated.

</TXT>

</DOC>

Below is the output of annotation (the changes made by annotator are highlighted). Note that the annotator has changed HF to “Y” in one case, to “N” in the second, and has added a third entry with the AF field set to “X”. In addition, the annotator has marked instances of each found gene with in-line XML in the text with the gene name tag (gn).

<DOC pmid="110315">

<DBGENELIST>

<DBGENE DBID="FBgn0000592" AF="Y" HF="Y"/><!--Est-6, Esterase 6, CG6917, Est-D, EST6, est-6, Est6, Est, EST-6, Esterase-6, est6, Est-5, Carboxyl ester hydrolase-->

<DBGENE DBID="FBgn0002722" AF="N" HF="N"/><!--m(Est-6), modifier of Esterase 6, M-est, m-est-->

<DBGENE DBID="FBgn0026412" AF=”X” HF="Y"/> <!-- leucine aminopeptidase -->

</DBGENELIST>

<TXT>

A locus has been found, an allele of which causes a modification of some

allozymes of the enzyme esterase 6 in Drosophila melanogaster. There are

two alleles of this locus, one of which is dominant to the other and

results in increased electrophoretic mobility of affected allozymes. The

locus responsible has been mapped to 3-56.7 on the standard genetic map

(<gn ID="FBgn0000592">Est-6</gn> is at 3-36.8). Of 13 other enzyme systems
analyzed, only <gn ID=” FBgn0026412”>leucine aminopeptidase</gn> is affected
by the modifier locus. Neuraminidase incubations of homogenates altered the
electrophoretic mobility of esterase 6 allozymes, but the mobility differences
found are not large enough to conclude that esterase 6 is sialylated.

</TXT>

</DOC>

Not all instances in the text need to be hand marked.. If the annotator knows what a gene is, but it is not listed exactly or not listed at all, they should tag it. Note, that alpha3beta1 integrin is tagged, because it probably is Itga3. Gene that is mentioned in the text that is not found in the list should be tagged as above. You do not need to tag a gene name if it is obvious what it is.

<DOC pmid="10069334">

<DBGENELIST>

<DBGENE DBID="MGI:101784" AF="N" HF="N"/><!-- Nes, nestin, ESTM46-->

<DBGENE DBID="MGI:96602" AF="N" HF="Y"/><!-- Itga3, integrin alpha 3, VLA-3 receptor, alpha 3 subunit, CD49C-->

<DBGENE DBID="MGI:96608" AF="N" HF="Y"/><!-- Itgav, integrin alpha V, vitronectin receptor alpha polypeptide (VNRA), CD51-->

</DBGENELIST>

<TXT>

Changes in specific cell-cell recognition and adhesion interactions

between neurons and radial glial cells regulate neuronal migration as well

as the establishment of distinct layers in the developing cerebral cortex.

Here, we show that <gn ID=”MGI:96602”>alpha3beta1 integrin</gn> is necessary for neuron-glial

recognition during neuronal migration and that <gn ID="MGI:96608">alpha(v) integrins</gn> provide

optimal levels of the basic neuron-glial adhesion needed to maintain

neuronal migration on radial glial fibers. A gliophilic-to-neurophilic

switch in the adhesive preference of developing cortical neurons occurs

following the loss of alpha3beta1 integrin function. Furthermore, the

targeted mutation of the <gn ID=”MGI:96602”>alpha3 integrin</gn> gene results in abnormal layering

of the cerebral cortex. These results suggest that alpha3beta1 and

alpha(v) integrins regulate distinct aspects of neuronal migration and

neuron-glial interactions during corticogenesis.

</TXT>

</DOC>

Aggregate members do not need to be tagged in the text (see 4b under Task). The hand annotated data will be provided in this format for a small sample of the training data (100-150 abstracts). Since synonyms are not unique, a gene name in the text might correspond to multiple genes. In these cases, both unique gene identifiers will be included. The annotator should make the correct gene, if they can.

<DOC pmid="9618507">

<DBGENELIST>

<DBGENE DBID="MGI:1346869" AF="Y" HF="Y"/><!-- Map2k4, mitogen activated protein kinase kinase 4, PRKMK4, Serk1, JNKK1, Sek1, MKK4, MEK4-->

<DBGENE DBID="MGI:98277" AF="Y" HF="N"/><!-- Epha4, Eph receptor A4, Tyro1, Sek1, Hek8, Cek8, Sek-->

<DBGENE DBID="MGI:1346862" AF="X" HF="Y"/><!-- Mapk9 mitogen activated protein kinase 9 JNK/SAPK alpha Jun kinase p54aSAPK Prkm9 JNK2-->

<DBGENE DBID="MGI:1346864" AF="X" HF="Y"/><!-- Mapk13 mitogen activated protein kinase 13 p38 delta MAP kinase Serk4-->

</DBGENELIST>

<TXT>

<gn ID="MGI:1346869 MGI:98277">SEK1</gn> (<gn ID="MGI:1346869">MKK4</gn>/JNKK) is a mitogen-activated protein kinase activator that has

been shown to participate in vitro in two stress-activated cascades

terminating with the SAPK and p38 kinases. To define the role of <gn ID="MGI:1346869 MGI:98277">SEK1</gn> in

vivo, we studied stress-induced signaling in <gn ID="MGI:1346869 MGI:98277">SEK1</gn>(-/-) embryonic stem and

fibroblast cells and evaluated the phenotype of <gn ID="MGI:1346869 MGI:98277">SEK1</gn>(-/-) mouse embryos

during development. Studies of <gn ID="MGI:1346869 MGI:98277">SEK1</gn>(-/-) embryonic stem cells demonstrated

defects in stimulated SAPK phosphorylation but not in the phosphorylation

of p38 kinase. In contrast, <gn ID="MGI:1346869 MGI:98277">SEK1</gn>(-/-) fibroblasts exhibited defects in

both SAPK and p38 phosphorylation, demonstrating that crosstalk exists

between the stress-activated cascades. Tumor necrosis factor alpha and

interleukin 1 stimulation of both stress-activated cascades are severely

affected in the <gn ID="MGI:1346869 MGI:98277">SEK1</gn>(-/-) fibroblast cells. <gn ID="MGI:1346869 MGI:98277">SEK1</gn> deficiency leads to

embryonic lethality after embryonic day 12.5 and is associated with

abnormal liver development. This phenotype is similar to c-jun null mouse

embryos and suggests that <gn ID="MGI:1346869 MGI:98277">SEK1</gn> is required for phosphorylation and

activation of c-jun during the organo-genesis of the liver.

</TXT>

</DOC>

These carefully annotated data could perhaps be used as a held out development test set.
